# Manganese(II) octa­uranium(IV) hepta­deca­sulfide

**DOI:** 10.1107/S1600536811030546

**Published:** 2011-08-06

**Authors:** George N. Oh, James A. Ibers

**Affiliations:** a Department of Chemistry, Northwestern University, 2145 Sheridan Rd., Evanston, IL 60208-3113, USA

## Abstract

Single crystals of manganese(II) octa­uranium(IV) hepta­deca­sulfide, MnU_8_S_17_, were grown from the reaction of the elements in a RbCl flux. MnU_8_S_17_ crystallizes in the space group *C*2/*m* in the CrU_8_S_17_ structure type. The asymmetric unit is composed of the following atoms with site symmetries shown: U1 (1), U2 (*m*), U3 (*m*), Mn1 (2/*m*), S1 (1), S2 (1); S3 (*m*), S4 (*m*), S5 (*m*), S6 (*m*) and S7 (2/*m*). The three U^IV^ atoms are each coordinated by eight S atoms in a bicapped trigonal–prismatic arrangement. The Mn^II^ atom is coordinated by six S atoms in a distorted octa­hedral arrangement.

## Related literature

MnU_8_S_17_ was previously determined to be isostructural with CrU_8_S_17_ (Noël *et al.*, 1975) ▶ from powder diffraction data (Noël, 1973 ▶). Single-crystal refinements have been carried out for the Cr, Fe (Kohlmann *et al.*, 1997 ▶), and Sc (Vovan & Rodier, 1979 ▶) analogues. Magnetic data for these compounds are available (Noël & Troc, 1979 ▶). The Mn—S distances are consistent with those expected for low-spin Mn^II^ (Shannon, 1976 ▶). For synthetic details, see: Bugaris & Ibers (2008 ▶); Haneveld & Jellinek (1969 ▶). For computational details, see: Gelato & Parthé (1987 ▶).

## Experimental

### 

#### Crystal data

MnU_8_S_17_
*M* *_r_* = 2504.20
Monoclinic, 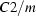

*a* = 13.3549 (6) Å
*b* = 8.3893 (4) Å
*c* = 10.4927 (5) Å
β = 101.658 (2)°
*V* = 1151.33 (9) Å^3^
*Z* = 2
Mo *K*α radiation
μ = 58.10 mm^−1^
*T* = 100 K
0.15 × 0.09 × 0.08 mm

#### Data collection

Bruker APEXII CCD diffractometer
Absorption correction: numerical face indexed (*SADABS*; Sheldrick, 2008*a* ▶) *T*_min_ = 0.043, *T*_max_ = 0.085
8422 measured reflections
1609 independent reflections
1578 reflections with *I* > 2σ(*I*)
*R*_int_ = 0.034

#### Refinement

*R*[*F* ^2^ > 2σ(*F* ^2^)] = 0.026
*wR*(*F* ^2^) = 0.068
*S* = 2.10
1609 reflections
73 parameters
Δρ_max_ = 3.90 e Å^−3^
Δρ_min_ = −1.94 e Å^−3^

### 

Data collection: *APEX2* (Bruker, 2009 ▶); cell refinement: *SAINT* (Bruker, 2009 ▶); data reduction: *SAINT*; program(s) used to solve structure: *SHELXS97* (Sheldrick, 2008*b* ▶); program(s) used to refine structure: *SHELXL97* (Sheldrick, 2008*b* ▶); molecular graphics: *CrystalMaker* (Palmer, 2009 ▶); software used to prepare material for publication: *SHELXL97*.

## Supplementary Material

Crystal structure: contains datablock(s) I, global. DOI: 10.1107/S1600536811030546/wm2512sup1.cif

Structure factors: contains datablock(s) I. DOI: 10.1107/S1600536811030546/wm2512Isup2.hkl

Additional supplementary materials:  crystallographic information; 3D view; checkCIF report

## Figures and Tables

**Fig. 1.**
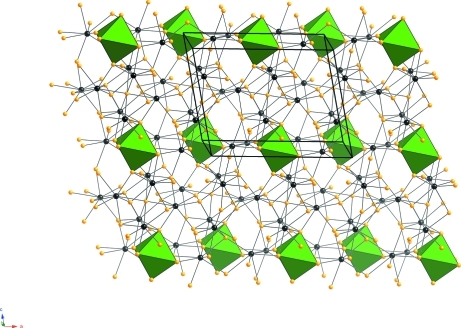
A packing diagram for MnU8S17, viewed nearly along the b axis. U atoms are black, S atoms are yellow, and the MnSe6 octahedra are green.

**Fig. 2.**
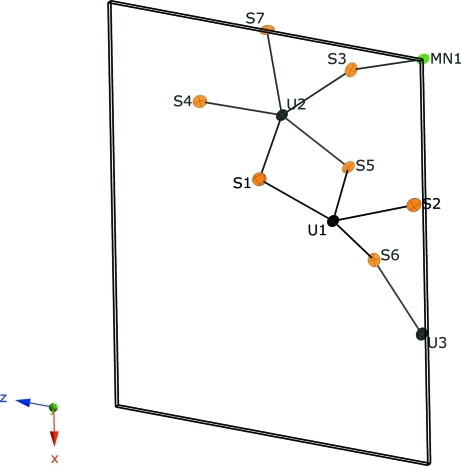
The asymmetric unit of MnU8S17. Displacement ellipsoids at the 95% probability level are shown.

## supplementary crystallographic information

### Comment

Single crystals of MnU_8_S_17_ were obtained in an attempt to synthesize a quaternary arsenic-containing compound (see Experimental).

MnU_8_S_17_ crystallizes in the CrU_8_S_17_ structure type (Noël *et al.*, 1975). Structure determinations based on single crystal data for isostructural ScU_8_S_17_ (Vovan & Rodier, 1979) and FeU_8_S_17_ (Kohlmann *et al.*, 1997) have also been reported, and MnU_8_S_17_ was also determined to be isostructural from powder diffraction experiments (Noël, 1973).

The structure comprises three independent U atoms, each coordinated by eight S atoms in a bicapped trigonal prismatic arrangement, and one independent Mn atom that is coordinated by six S atoms in a distorted octahedral arrangement (Figs. 1,2). There are no S—S bonds in the structure, so formal oxidation states of +IV, +II, and -II may be assigned to U, Mn, and S, respectively. U—S distances have the following ranges: U1—S: 2.7546 (19) Å to 2.9509 (3) Å; U2—S: 2.735 (3) Å to 2.8559 (4) Å; U3—S: 2.680 (2) Å to 3.031 (3) Å. These distances are similar to those found in the structures of the Cr and Fe analogues. Mn—S distances range from 2.398 (3) Å to 2.5168 (19) Å; these are shorter than a typical high-spin six-coordinate Mn^II^—S distance of 2.66 Å, but are consistent with the typical low-spin six-coordinate Mn^II^—S distance of 2.51 Å (Shannon, 1976). Low-spin Mn^II^ is consistent with the ^6^S_5/2_ configuration assigned from magnetic studies (Noël & Troc, 1979).

### Experimental

Black blocks of MnU_8_S_17_ were obtained by combining U (0.126 mmol), Mn (Johnson Matthey 99.3%, 0.126 mmol), and S (Mallinckrodt 99.6% sublimed, 0.504 mmol) in a RbCl flux (Alfa 99.8%, 1.26 mmol) with As as a mineralizer (Strem 2N, 0.126 mmol). U filings (Oak Ridge National Laboratory) were powdered by hydridization and subsequent decomposition under heat and vacuum (Bugaris & Ibers, 2008), in a modification of a previous literature method (Haneveld & Jellinek, 1969). The mixture was loaded into a carbon-coated fused-silica tube in an Ar filled glove box and then sealed under 10 ^-4^ Torr vacuum. The vessel was heated in a computer-controlled furnace to 1073 K in 96 h, held for 96 h, cooled to 673 K in 96 h, then cooled to 298 K in 48 h. The flux was washed off with water and surface impurities were mechanically removed from a single crystal.

### Refinement

The structure was standardized by means of the program *STRUCTURE TIDY* (Gelato & Parthé, 1987). The highest peak of 3.9 (4) e/Å^3^ is 1.33 Å from atom U2 and the deepest hole of -1.9 (4) e/Å^3^ is 0.88 Å from atom U3.

### Crystal data

**Table:**

MnU_8_S_17_	*F*(000) = 2066
*M**_r_* = 2504.20	*D*_x_ = 7.223 Mg m^−^^3^
Monoclinic, *C*2/*m*	Mo *K*α radiation, λ = 0.71073 Å
Hall symbol: -C 2y	Cell parameters from 8717 reflections
*a* = 13.3549 (6) Å	θ = 2.9–30.5°
*b* = 8.3893 (4) Å	µ = 58.10 mm^−^^1^
*c* = 10.4927 (5) Å	*T* = 100 K
β = 101.658 (2)°	Rectangular block, black
*V* = 1151.33 (9) Å^3^	0.15 × 0.09 × 0.08 mm
*Z* = 2	

### Data collection

**Table:**

Bruker APEXII CCD diffractometer	1609 independent reflections
Radiation source: fine-focus sealed tube	1578 reflections with *I* > 2σ(*I*)
graphite	*R*_int_ = 0.034
φ and ω scans	θ_max_ = 29.1°, θ_min_ = 2.0°
Absorption correction: numerical face indexed (*SADABS*; Sheldrick, 2008*a*)	*h* = −18→17
*T*_min_ = 0.043, *T*_max_ = 0.085	*k* = −10→11
8422 measured reflections	*l* = −14→14

### Refinement

**Table:**

Refinement on *F*^2^	Primary atom site location: structure-invariant direct methods
Least-squares matrix: full	Secondary atom site location: difference Fourier map
*R*[*F*^2^ > 2σ(*F*^2^)] = 0.026	*w* = 1/[σ^2^(*F*_o_^2^) + (0.0161*F*_o_^2^)^2^]
*wR*(*F*^2^) = 0.068	(Δ/σ)_max_ = 0.001
*S* = 2.10	Δρ_max_ = 3.90 e Å^−^^3^
1609 reflections	Δρ_min_ = −1.94 e Å^−^^3^
73 parameters	Extinction correction: *SHELXL97* (Sheldrick, 2008a), Fc^*^=kFc[1+0.001xFc^2^λ^3^/sin(2θ)]^-1/4^
0 restraints	Extinction coefficient: 0.00082 (4)

### Fractional atomic coordinates and isotropic or equivalent isotropic displacement parameters (Å^2^)

**Table:**

	*x*	*y*	*z*	*U*_iso_*/*U*_eq_	
U1	0.44179 (2)	0.25549 (3)	0.29633 (3)	0.00531 (11)	
U2	0.20440 (3)	0.0000	0.45685 (4)	0.00510 (12)	
U3	0.68285 (3)	0.0000	0.02025 (4)	0.00541 (12)	
Mn1	0.0000	0.0000	0.0000	0.0058 (5)	
S1	0.12730 (15)	0.3047 (2)	0.46870 (18)	0.0065 (4)	
S2	0.36424 (15)	0.3062 (2)	0.03492 (18)	0.0064 (4)	
S3	0.0598 (2)	0.0000	0.2314 (3)	0.0066 (5)	
S4	0.2086 (2)	0.0000	0.7197 (3)	0.0061 (5)	
S5	0.3030 (2)	0.0000	0.2463 (3)	0.0058 (5)	
S6	0.5211 (2)	0.0000	0.1694 (3)	0.0061 (5)	
S7	0.0000	0.0000	0.5000	0.0056 (7)	

### Atomic displacement parameters (Å^2^)

**Table:**

	*U*^11^	*U*^22^	*U*^33^	*U*^12^	*U*^13^	*U*^23^
U1	0.0056 (2)	0.00542 (18)	0.00528 (17)	−0.00005 (10)	0.00195 (13)	0.00004 (10)
U2	0.0053 (2)	0.0054 (2)	0.0051 (2)	0.000	0.00217 (15)	0.000
U3	0.0063 (2)	0.0052 (2)	0.00499 (19)	0.000	0.00170 (16)	0.000
Mn1	0.0047 (12)	0.0068 (11)	0.0064 (10)	0.000	0.0024 (9)	0.000
S1	0.0062 (10)	0.0067 (9)	0.0069 (8)	−0.0004 (8)	0.0017 (7)	0.0000 (7)
S2	0.0064 (10)	0.0058 (9)	0.0075 (8)	−0.0008 (7)	0.0023 (7)	0.0000 (7)
S3	0.0079 (14)	0.0067 (13)	0.0057 (11)	0.000	0.0024 (10)	0.000
S4	0.0059 (14)	0.0056 (12)	0.0071 (12)	0.000	0.0018 (10)	0.000
S5	0.0059 (14)	0.0062 (12)	0.0058 (11)	0.000	0.0027 (10)	0.000
S6	0.0067 (14)	0.0059 (12)	0.0055 (12)	0.000	0.0006 (10)	0.000
S7	0.0034 (19)	0.0050 (16)	0.0091 (17)	0.000	0.0025 (14)	0.000

### Geometric parameters (Å, °)

**Table:**

U1—S3^i^	2.7546 (19)	U3—U1^iv^	4.0205 (4)
U1—S2	2.7621 (19)	U3—U1^vii^	4.0205 (4)
U1—S1^ii^	2.802 (2)	Mn1—S3	2.398 (3)
U1—S5	2.8129 (19)	Mn1—S3^x^	2.398 (3)
U1—S6	2.8390 (18)	Mn1—S2^xi^	2.5168 (19)
U1—S1^iii^	2.8469 (19)	Mn1—S2^xii^	2.5168 (19)
U1—S4^iii^	2.852 (2)	Mn1—S2^xiii^	2.5168 (19)
U1—S7^i^	2.9509 (3)	Mn1—S2^xiv^	2.5168 (19)
U1—U3^iv^	4.0206 (4)	S1—U2^iii^	2.763 (2)
U1—U2^iii^	4.0951 (5)	S1—U1^xiv^	2.802 (2)
U2—S3	2.735 (3)	S1—U1^iii^	2.8469 (19)
U2—S4	2.747 (3)	S2—Mn1^i^	2.5168 (19)
U2—S1^iii^	2.763 (2)	S2—U3^iv^	2.680 (2)
U2—S1^v^	2.763 (2)	S2—U3^xv^	2.896 (2)
U2—S1	2.768 (2)	S3—U1^xi^	2.7546 (19)
U2—S1^vi^	2.768 (2)	S3—U1^xiv^	2.7546 (19)
U2—S5	2.791 (3)	S4—U3^viii^	2.819 (3)
U2—S7	2.8559 (4)	S4—U1^v^	2.852 (2)
U2—U1^iii^	4.0952 (5)	S4—U1^iii^	2.852 (2)
U2—U1^v^	4.0952 (5)	S5—U1^vi^	2.8130 (19)
U3—S2^iv^	2.680 (2)	S5—U3^iv^	2.841 (3)
U3—S2^vii^	2.680 (2)	S6—U1^vi^	2.8390 (18)
U3—S4^viii^	2.819 (3)	S6—U3^iv^	3.031 (3)
U3—S5^iv^	2.841 (3)	S7—U2^xvi^	2.8559 (4)
U3—S2^ii^	2.896 (2)	S7—U1^xi^	2.9509 (3)
U3—S2^ix^	2.896 (2)	S7—U1^iii^	2.9509 (3)
U3—S6	2.912 (3)	S7—U1^v^	2.9509 (3)
U3—S6^iv^	3.031 (3)	S7—U1^xiv^	2.9509 (3)
			
S3^i^—U1—S2	76.01 (7)	S2^vii^—U3—S2^ix^	134.99 (5)
S3^i^—U1—S1^ii^	79.55 (7)	S4^viii^—U3—S2^ix^	71.79 (6)
S2—U1—S1^ii^	140.70 (6)	S5^iv^—U3—S2^ix^	80.27 (6)
S3^i^—U1—S5	155.43 (8)	S2^ii^—U3—S2^ix^	68.33 (8)
S2—U1—S5	80.28 (7)	S2^iv^—U3—S6	87.07 (5)
S1^ii^—U1—S5	116.52 (6)	S2^vii^—U3—S6	87.07 (5)
S3^i^—U1—S6	99.20 (7)	S4^viii^—U3—S6	76.83 (8)
S2—U1—S6	75.58 (7)	S5^iv^—U3—S6	137.15 (8)
S1^ii^—U1—S6	78.56 (7)	S2^ii^—U3—S6	132.44 (5)
S5—U1—S6	68.37 (7)	S2^ix^—U3—S6	132.44 (5)
S3^i^—U1—S1^iii^	130.39 (7)	S2^iv^—U3—S6^iv^	73.60 (4)
S2—U1—S1^iii^	139.93 (6)	S2^vii^—U3—S6^iv^	73.60 (4)
S1^ii^—U1—S1^iii^	78.93 (6)	S4^viii^—U3—S6^iv^	148.59 (8)
S5—U1—S1^iii^	73.14 (7)	S5^iv^—U3—S6^iv^	65.38 (8)
S6—U1—S1^iii^	119.29 (6)	S2^ii^—U3—S6^iv^	131.75 (5)
S3^i^—U1—S4^iii^	83.16 (6)	S2^ix^—U3—S6^iv^	131.75 (5)
S2—U1—S4^iii^	73.29 (7)	S6—U3—S6^iv^	71.77 (8)
S1^ii^—U1—S4^iii^	133.46 (7)	S2^iv^—U3—U1^iv^	43.17 (4)
S5—U1—S4^iii^	96.17 (6)	S2^vii^—U3—U1^iv^	107.02 (4)
S6—U1—S4^iii^	147.19 (8)	S4^viii^—U3—U1^iv^	147.748 (6)
S1^iii^—U1—S4^iii^	80.27 (7)	S5^iv^—U3—U1^iv^	44.40 (4)
S3^i^—U1—S7^i^	65.25 (5)	S2^ii^—U3—U1^iv^	123.06 (4)
S2—U1—S7^i^	127.10 (4)	S2^ix^—U3—U1^iv^	86.95 (4)
S1^ii^—U1—S7^i^	65.71 (4)	S6—U3—U1^iv^	102.46 (5)
S5—U1—S7^i^	137.07 (6)	S6^iv^—U3—U1^iv^	44.81 (3)
S6—U1—S7^i^	142.74 (6)	S2^iv^—U3—U1^vii^	107.02 (4)
S1^iii^—U1—S7^i^	65.18 (4)	S2^vii^—U3—U1^vii^	43.17 (4)
S4^iii^—U1—S7^i^	67.79 (5)	S4^viii^—U3—U1^vii^	147.748 (6)
S3^i^—U1—U3^iv^	110.77 (6)	S5^iv^—U3—U1^vii^	44.40 (4)
S2—U1—U3^iv^	41.58 (4)	S2^ii^—U3—U1^vii^	86.95 (4)
S1^ii^—U1—U3^iv^	126.98 (4)	S2^ix^—U3—U1^vii^	123.06 (4)
S5—U1—U3^iv^	44.95 (6)	S6—U3—U1^vii^	102.46 (5)
S6—U1—U3^iv^	48.80 (6)	S6^iv^—U3—U1^vii^	44.81 (3)
S1^iii^—U1—U3^iv^	117.93 (4)	U1^iv^—U3—U1^vii^	64.432 (10)
S4^iii^—U1—U3^iv^	99.54 (5)	S3—Mn1—S3^x^	180.0
S7^i^—U1—U3^iv^	166.765 (10)	S3—Mn1—S2^xi^	87.41 (7)
S3^i^—U1—U2^iii^	98.80 (5)	S3^x^—Mn1—S2^xi^	92.59 (7)
S2—U1—U2^iii^	114.83 (4)	S3—Mn1—S2^xii^	92.59 (7)
S1^ii^—U1—U2^iii^	98.88 (4)	S3^x^—Mn1—S2^xii^	87.41 (7)
S5—U1—U2^iii^	96.86 (5)	S2^xi^—Mn1—S2^xii^	180.00 (11)
S6—U1—U2^iii^	161.01 (4)	S3—Mn1—S2^xiii^	92.59 (7)
S1^iii^—U1—U2^iii^	42.42 (4)	S3^x^—Mn1—S2^xiii^	87.41 (7)
S4^iii^—U1—U2^iii^	42.01 (5)	S2^xi^—Mn1—S2^xiii^	99.50 (9)
S7^i^—U1—U2^iii^	44.218 (7)	S2^xii^—Mn1—S2^xiii^	80.50 (9)
U3^iv^—U1—U2^iii^	128.230 (12)	S3—Mn1—S2^xiv^	87.41 (7)
S3—U2—S4	137.38 (8)	S3^x^—Mn1—S2^xiv^	92.59 (7)
S3—U2—S1^iii^	129.53 (6)	S2^xi^—Mn1—S2^xiv^	80.50 (9)
S4—U2—S1^iii^	82.29 (6)	S2^xii^—Mn1—S2^xiv^	99.50 (9)
S3—U2—S1^v^	129.53 (6)	S2^xiii^—Mn1—S2^xiv^	180.00 (9)
S4—U2—S1^v^	82.29 (6)	U2^iii^—S1—U2	105.75 (7)
S1^iii^—U2—S1^v^	72.72 (8)	U2^iii^—S1—U1^xiv^	148.40 (8)
S3—U2—S1	80.49 (5)	U2—S1—U1^xiv^	95.33 (6)
S4—U2—S1	83.52 (5)	U2^iii^—S1—U1^iii^	104.35 (6)
S1^iii^—U2—S1	74.25 (7)	U2—S1—U1^iii^	93.65 (6)
S1^v^—U2—S1	145.44 (5)	U1^xiv^—S1—U1^iii^	97.35 (6)
S3—U2—S1^vi^	80.49 (5)	Mn1^i^—S2—U3^iv^	137.09 (8)
S4—U2—S1^vi^	83.52 (5)	Mn1^i^—S2—U1	96.17 (6)
S1^iii^—U2—S1^vi^	145.44 (5)	U3^iv^—S2—U1	95.25 (6)
S1^v^—U2—S1^vi^	74.25 (7)	Mn1^i^—S2—U3^xv^	104.41 (7)
S1—U2—S1^vi^	134.89 (9)	U3^iv^—S2—U3^xv^	111.68 (7)
S3—U2—S5	71.28 (8)	U1—S2—U3^xv^	106.32 (6)
S4—U2—S5	151.35 (9)	Mn1—S3—U2	155.27 (12)
S1^iii^—U2—S5	74.75 (6)	Mn1—S3—U1^xi^	99.23 (8)
S1^v^—U2—S5	74.75 (6)	U2—S3—U1^xi^	97.20 (7)
S1—U2—S5	105.95 (4)	Mn1—S3—U1^xiv^	99.23 (8)
S1^vi^—U2—S5	105.95 (4)	U2—S3—U1^xiv^	97.20 (7)
S3—U2—S7	66.83 (6)	U1^xi^—S3—U1^xiv^	96.26 (9)
S4—U2—S7	70.55 (6)	U2—S4—U3^viii^	150.90 (11)
S1^iii^—U2—S7	134.75 (4)	U2—S4—U1^v^	93.99 (7)
S1^v^—U2—S7	134.75 (4)	U3^viii^—S4—U1^v^	105.98 (7)
S1—U2—S7	67.46 (4)	U2—S4—U1^iii^	93.99 (7)
S1^vi^—U2—S7	67.46 (4)	U3^viii^—S4—U1^iii^	105.98 (7)
S5—U2—S7	138.10 (6)	U1^v^—S4—U1^iii^	91.99 (8)
S3—U2—U1^iii^	101.73 (5)	U2—S5—U1	104.51 (7)
S4—U2—U1^iii^	44.00 (4)	U2—S5—U1^vi^	104.51 (7)
S1^iii^—U2—U1^iii^	89.36 (4)	U1—S5—U1^vi^	99.28 (9)
S1^v^—U2—U1^iii^	125.64 (4)	U2—S5—U3^iv^	156.22 (12)
S1—U2—U1^iii^	43.93 (4)	U1—S5—U3^iv^	90.65 (7)
S1^vi^—U2—U1^iii^	101.83 (4)	U1^vi^—S5—U3^iv^	90.65 (7)
S5—U2—U1^iii^	149.546 (11)	U1^vi^—S6—U1	98.05 (8)
S7—U2—U1^iii^	46.101 (7)	U1^vi^—S6—U3	129.76 (5)
S3—U2—U1^v^	101.73 (5)	U1—S6—U3	129.76 (5)
S4—U2—U1^v^	44.00 (4)	U1^vi^—S6—U3^iv^	86.39 (7)
S1^iii^—U2—U1^v^	125.64 (4)	U1—S6—U3^iv^	86.39 (7)
S1^v^—U2—U1^v^	89.36 (4)	U3—S6—U3^iv^	108.23 (8)
S1—U2—U1^v^	101.83 (4)	U2—S7—U2^xvi^	180.0
S1^vi^—U2—U1^v^	43.93 (4)	U2—S7—U1^xi^	90.317 (8)
S5—U2—U1^v^	149.546 (10)	U2^xvi^—S7—U1^xi^	89.682 (8)
S7—U2—U1^v^	46.101 (7)	U2—S7—U1^iii^	89.683 (8)
U1^iii^—U2—U1^v^	60.118 (10)	U2^xvi^—S7—U1^iii^	90.318 (8)
S2^iv^—U3—S2^vii^	146.88 (9)	U1^xi^—S7—U1^iii^	180.0
S2^iv^—U3—S4^viii^	105.15 (4)	U2—S7—U1^v^	89.683 (8)
S2^vii^—U3—S4^viii^	105.15 (4)	U2^xvi^—S7—U1^v^	90.318 (8)
S2^iv^—U3—S5^iv^	81.18 (4)	U1^xi^—S7—U1^v^	91.925 (11)
S2^vii^—U3—S5^iv^	81.18 (4)	U1^iii^—S7—U1^v^	88.075 (11)
S4^viii^—U3—S5^iv^	146.02 (8)	U2—S7—U1^xiv^	90.317 (8)
S2^iv^—U3—S2^ii^	134.99 (5)	U2^xvi^—S7—U1^xiv^	89.682 (8)
S2^vii^—U3—S2^ii^	68.31 (7)	U1^xi^—S7—U1^xiv^	88.075 (11)
S4^viii^—U3—S2^ii^	71.79 (6)	U1^iii^—S7—U1^xiv^	91.925 (11)
S5^iv^—U3—S2^ii^	80.27 (6)	U1^v^—S7—U1^xiv^	180.0
S2^iv^—U3—S2^ix^	68.31 (7)		

Symmetry codes: (i) *x*+1/2, *y*+1/2, *z*; (ii) *x*+1/2, −*y*+1/2, *z*; (iii) −*x*+1/2, −*y*+1/2, −*z*+1; (iv) −*x*+1, −*y*, −*z*; (v) −*x*+1/2, *y*−1/2, −*z*+1; (vi) *x*, −*y*, *z*; (vii) −*x*+1, *y*, −*z*; (viii) −*x*+1, −*y*, −*z*+1; (ix) *x*+1/2, *y*−1/2, *z*; (x) −*x*, −*y*, −*z*; (xi) *x*−1/2, *y*−1/2, *z*; (xii) −*x*+1/2, −*y*+1/2, −*z*; (xiii) −*x*+1/2, *y*−1/2, −*z*; (xiv) *x*−1/2, −*y*+1/2, *z*; (xv) *x*−1/2, *y*+1/2, *z*; (xvi) −*x*, −*y*, −*z*+1.
